# Structure determination of the tetracene dimer in helium nanodroplets using femtosecond strong-field ionization

**DOI:** 10.1063/1.5118005

**Published:** 2019-08-27

**Authors:** Constant Schouder, Adam S. Chatterley, Florent Calvo, Lars Christiansen, Henrik Stapelfeldt

**Affiliations:** 1 Department of Physics and Astronomy, Aarhus University, Ny Munkegade 120, 8000 Aarhus C, Denmark; 2 Department of Chemistry, Aarhus University, Langelandsgade 140, 8000 Aarhus C, Denmark; 3 Université Grenoble Alpes CNRS, LIPHY, F-38000 Grenoble, France

## Abstract

Dimers of tetracene molecules are formed inside helium nanodroplets and identified through covariance analysis of the emission directions of kinetic tetracene cations stemming from femtosecond laser-induced Coulomb explosion. Next, the dimers are aligned in either one or three dimensions under field-free conditions by a nonresonant, moderately intense laser pulse. The experimental angular covariance maps of the tetracene ions are compared to calculated covariance maps for seven different dimer conformations and found to be consistent with four of these. Additional measurements of the alignment-dependent strong-field ionization yield of the dimer narrow the possible conformations down to either a slipped-parallel or parallel-slightly rotated structure. According to our quantum chemistry calculations, these are the two most stable gas-phase conformations of the dimer and one of them is favorable for singlet fission.

## I. INTRODUCTION

Noncovalent interactions between aromatic molecules are crucial for many areas, such as molecular recognition, structure of macromolecules, and organic solar cells.^1–4^ At the most fundamental level, the interaction involves two aromatic molecules. This has been the subject of a large numbers of studies, often with a particular focus on determining the structure of the dimers. Experimentally, the main technique to form dimers is supersonic expansion of a molecular gas seeded in a carrier gas of rare gas atoms into vacuum. Combining the resulting molecular beams with various types of high-resolution spectroscopy, including microwave, infrared, and UV spectroscopy^3,5,6^ as well as rotational coherence spectroscopy^7,8^—a technique based on pairs of femtosecond or picosecond pulses—the rotational constants can be extracted. Upon comparison with results from theoretical modeling, information about the conformations of a range of different dimers have been obtained.^3,9^ Examples include the dimers of benzene,^10^ fluorene,^11^ benzonitrile,^12,13^ phenol,^14^ and anisole.^15^

An alternative experimental method is to form molecular dimers or larger oligomers inside helium nanodroplets.^16,17^ This technique makes it possible to create aggregates of much larger molecules, for instance of fullerenes^18^ or polycyclic aromatic hydrocarbons (PAHs),^19–21^ than what is typically possible in molecular beams using standard supersonic expansions. Furthermore, the variety of heterogenous aggregates goes beyond the normal reach of the gas phase.^16,17^ Structure characterization has mainly been obtained by IR spectroscopy^22–24^ although for complexes of larger molecules, this becomes very challenging, as the density of states is too high to clearly resolve peaks in the spectra.

Recently, we introduced an alternative method for structure determination of dimers created inside He droplets, namely, Coulomb explosion induced by an intense femtosecond (fs) laser pulse and recording of the emission direction of the fragment ions including identification of their angular correlations,^25,26^ implemented through covariance analysis.^27–29^ Crucial to the structure determination was that the dimers had a well-defined spatial orientation prior to the Coulomb explosion event, in practice obtained by laser-induced alignment with a moderately intense laser pulse.^30,31^ While the Coulomb explosion method may not match the level of structural accuracy possible with high resolution spectroscopy, at least not for comparatively small molecules, it distinguishes itself by the fact that the structure is captured within the time scale of the pulse duration, i.e., in less than 50 fs. As such, this technique holds the potential for imaging the structure of dimers as they undergo rapid structural change, for example, due to excimer formation. The purpose of the current manuscript is to show that the Coulomb explosion method can also be used to obtain structure information about dimers composed of molecules much larger than the carbon disulfide and carbonyl sulfide molecules studied so far.^25,26^ Here, we explore the dimer of tetracene (Tc), a polycyclic aromatic hydrocarbon (PAH) composed of four linearly fused benzene rings. We demonstrate that a covariance map analysis of the angular distributions of fragments from fs laser-induced Coulomb explosion supplemented by the measurement of alignment-dependent ion yields allows us to identify the tetracene conformation as a face-to-face structure with the tetracene monomers either slightly displaced or slightly rotated. This identification relies on comparison of the experimental covariance maps to simulated covariance maps for a range of plausible conformations.

Our motivation for exploring the structure of the dimer is threefold. First, PAHs are known to be relatively abundant in the interstellar medium,^32^ and laboratory experiments are required to deduce the signatures that astronomers will need to hunt for. Second, PAH oligomers of similar size such as the pyrene dimer have a possibly key role in the formation of soot,^33^ and it is important to characterize their structure and bonding with accuracy. Third, noncovalently bonded ensembles of tetracene and other acenes can undergo singlet fission,^4,34^ a phenomenon with major implications for solar energy harvesting where a singlet excited state decays into two triplet states localized on two separate monomers. The process produces two excitations from a single photon, which in principle provides a means to overcome the Shockley-Queisser 33% efficiency limit inherent to single excitation photovoltaic systems. Typically, singlet fission is studied in crystals and thin films, which are excellent for emulating photovoltaic devices but due to their extended nature are less well suited for investigating the basic photophysical mechanisms, and so fundamental understandings of singlet fission have somewhat lagged behind technological developments. Solution studies of carefully synthesized covalent dimer systems with only two acene units provide clearer insight into the fundamental dynamics, with the caveat that the covalent linkage may interfere with the electronic structure, and solvent and temperature effects blur spectra.^35,36^ Helium nanodroplets allow formation of the pristine dimers without the need for an extended system or any covalent bond. Structure determination is essential as the relative configuration of the monomer in a polyacene dimer is crucial for the singlet fission process: the *π* systems must overlap, but there must also be an offset between the two units for singlet fission to be an allowed process.^4,34^ The conformations observed in the present work are favorable for singlet fission, paving the way for future time-resolved studies of singlet fission processes in controlled environments.

## II. EXPERIMENTAL SETUP

**FIG. 1.**
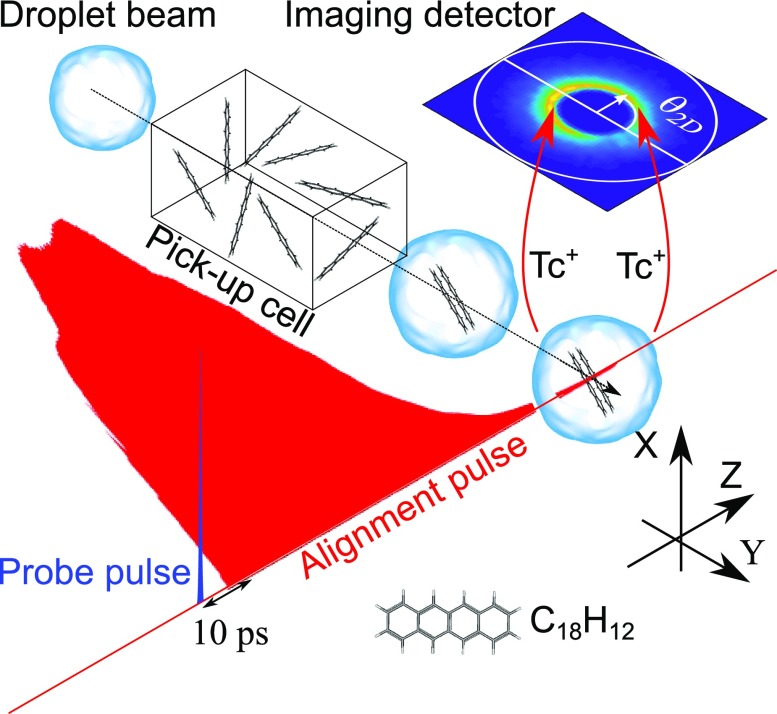
Schematic of the key elements in the experiment. Tc dimers are first formed inside He droplets and then aligned by a truncated nonresonant alignment laser pulse (red). 10 ps after truncation of the alignment pulse, the Tc dimers are doubly ionized by a short intense probe pulse (blue) to induce Coulomb explosion and the velocities of the two Tc^+^ ions are measured using velocity map imaging. In all measurements, the probe pulse was linearly polarized along the X axis (perpendicular to the detector plane), whereas the alignment pulse was either linearly polarized along the X axis or the Y axis (depicted here) or elliptically polarized with the major polarization along the X axis or the Y axis.

The experimental setup has been described in detail before,^37^ and only important aspects are pointed out here. A schematic of the setup is shown in Fig. 1. A continuous beam of He droplets is formed by expanding He gas through a 5 *μ*m nozzle, cooled to 12 K, into vacuum. The backing pressure is 25 bar, leading to droplets consisting on average of 10 000 He atoms.^38^ The droplets then pass through a pickup cell containing tetracene vapor obtained by resistively heating a sample of solid tetracene. The probability for a droplet to pick up one or two Tc molecules depends on the partial pressure of tetracene in the cell^39^ defined by its temperature. As discussed in Sec. IV, this allows us to control the formation of Tc dimers inside the He droplets.

Hereafter, the doped He droplet beam enters the target region where it is crossed, at right angles, by two collinear, focused, pulsed laser beams. The pulses in the first beam are used to induce alignment of the Tc dimers (and monomers) in the He droplets. They have an asymmetric temporal shape rising to a peak in ∼120 ps and turning-off in ∼10 ps—see sketch in Fig. 1 and measured intensity profile in Figs. 5 and 6—obtained by spectrally truncating the uncompressed pulses from an amplified Ti:sapphire laser system.^40^ Their central wavelength is 800 nm, the focal spot size, *ω*_0_, is 65 *μ*m, and the peak intensity is ∼0.16 TW/cm^2^. The pulses in the second beam are used to identify the formation of Tc dimers and measure their alignment. As detailed in Sec. IV, this relies on ionization of the Tc dimers. These probe pulses (35 fs long, *λ*_central_ = 400 nm) are created by second harmonic generation of the compressed output from the Ti:sapphire laser system in a 50 *μ*m thick Beta Barium Borate (BBO) crystal. The focal spot size, *ω*_0_, is 50 *μ*m and the intensity is varied from 0.6 to 9 TW/cm^2^. The repetition rate of the laser pulses is 1 kHz.

The Tc^+^ ions created by the probe pulse are projected by a velocity map imaging spectrometer onto a 2D imaging detector. The detector consists of two microchannel plates backed by a P47 phosphor screen, whose images are recorded on a Charge-coupled device (CCD) camera. The CCD camera is readout every 10 laser shots, i.e., at a 100 Hz rate, and such an image is termed a frame. Ion images as the ones shown in Fig. 3 typically consist of 10 000 frames.

## III. MOLECULAR MODELING OF THE TETRACENE DIMER CONFORMATION

The conformations of the tetracene dimer were independently explored by molecular modeling and quantum chemical methods in order to identify plausible candidates for interpreting the measurements.

**FIG. 2.**
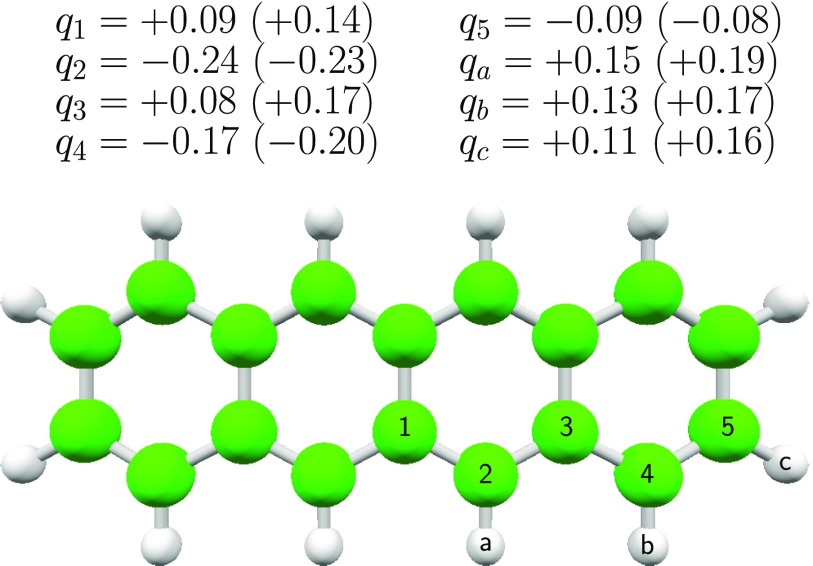
Partial charges on carbon and hydrogen atoms on the neutral tetracene monomer, as used in the force field exploration of dimer conformations. Partial charges for the tetracene cation used in the simulations are given in parentheses. All charges are expressed in units of the electron charge.

The potential energy landscape was first explored using a simple quantum mechanical model previously developed to simulate the sticking between PAH molecules under astrophysical conditions.^41^ Briefly, the model consists of an additive potential with intramolecular contributions *V*_intra_ for each tetracene molecule and a pairwise force field *V*_inter_ describing the noncovalent interactions between the two flexible molecules. Here, *V*_intra_ is based on an earlier tight-binding model of Van-Oanh and coworkers,^42^ while *V*_inter_ is a simple sum of Lennard-Jones (LJ) and Coulomb terms acting between the atomic positions that also carry partial charges representing the multipolar distribution. For the LJ potential, we employed two parameter sets either from the optimized potentials for liquid simulations (OPLS) library^43^ or published earlier by van de Waal in the context of hydrocarbon clusters.^44^ The partial charges on tetracene were evaluated using the fluctuating charge method,^45^ as proposed by Rapacioli and coworkers^46^ who adjusted its parameters so that it mimics the restrained electrostatic potential (RESP) procedure often used to extract charges from density-functional theory (DFT) calculations. The charges obtained for the tetracene monomer are shown in Fig. 2.

Our initial scanning procedure consisted of a large amplitude Monte Carlo exploration of the possible conformations of the dimer, followed by systematic local optimization using this flexible potential. The optimized geometries were then refined directly with quantum chemical methods, employing here again DFT with functionals that account for long-range (noncovalent) forces that are essential for the present system. The two functionals B97–1 and wB97xD were thus employed with the two basis sets 6–311G(d,p) and TZVP. Basis set superposition errors were accounted for using the standard counterpoise method, and from the equilibrium geometries, the harmonic zero-point energies were also evaluated. All quantum chemical calculations were performed using Gaussian09.^47^

Our exploration leads to only two locally stable conformations, with the two molecular planes parallel to each other, the main axes of the molecules being themselves either parallel as well or forming an angle of about 25°. In the former case, the molecules are not superimposed on each other but shifted in order to maximize van der Waals interactions (as in graphite). Consistent with standard terminology,^46^ the resulting conformations, numbered as 1 and 2 in Fig. 4, are referred to as parallel displaced or rotated, respectively, but we will also describe them as slipped-parallel and rotated-parallel. The other conformations shown in Fig. 4, numbered 3–7, are not locally stable with any of the methods used and relax into either of the two parallel conformers.

The relative energies of the two conformers are compared in Table I, and the binding energy of the most stable one is provided as well. From this table, we find a significant spreading in the values of the binding energy, which roughly varies from 200 meV for the B97–1 DFT method to 800 meV for the wB97xD method, the empirical models yielding values of about 500 meV in between these two extremes. However, the energy difference between the conformers appears as a fraction of the absolute binding energy whatever the level of calculation. From the perspective of the quantum force field, the two conformers are nearly isoenergetic, with the parallel displaced isomer being higher by less than 10 meV. The DFT results generally predict the same ordering, but with a slightly higher difference closer to 20–30 meV depending on whether zero-point correction is included or not, still quite small. Only the B97–1 functional with the TZVP basis set finds otherwise that the rotated conformer should not be the most stable of the two. At this stage, we cautiously conclude that two particular conformations for the tetracene dimer are candidates for experimental elucidation, both having the molecules parallel to each other but some shift or rotation between their main symmetry axes.

**TABLE I.** Binding energies and relative energies of the parallel displaced and rotated conformers of the tetracene dimer, as obtained from a simple quantum mechanical force field (TB+LJ) with two sets of LJ parameters or from density-functional theory minimizations with two functionals and two basis sets and after correcting for basis set superposition error. Absolute numbers shown in bold face represent the binding energies obtained for the most stable conformer, and numbers with a plus sign indicate the relative difference of the less stable conformer. The values in parentheses include the harmonic zero-point energy corrections. All values are given in milli-electron-volts.

Method	Parallel displaced	Rotated
TB+LJ (vdW)	+4.9 (+6.5)	**582.0 (554.1)**
TB+LJ (OPLS)	+6.8 (+8.1)	**437.5 (416.7)**
B97-1/6-311G(d,p)	+3.3 (+4.6)	**156.1 (148.6)**
B97-1/TZVP	**116.3 (100.4)**	+36.5 (+25.6)
wB97xD/6-311G(d,p)	+26.6 (+28.1)	**893.4 (868.1)**
wB97xD/TZVP	+19.9 (+16.7)	**730.7 (698.9)**

## IV. RESULTS: COULOMB EXPLOSION

### A. Identification of tetracene dimers

**FIG. 3.**
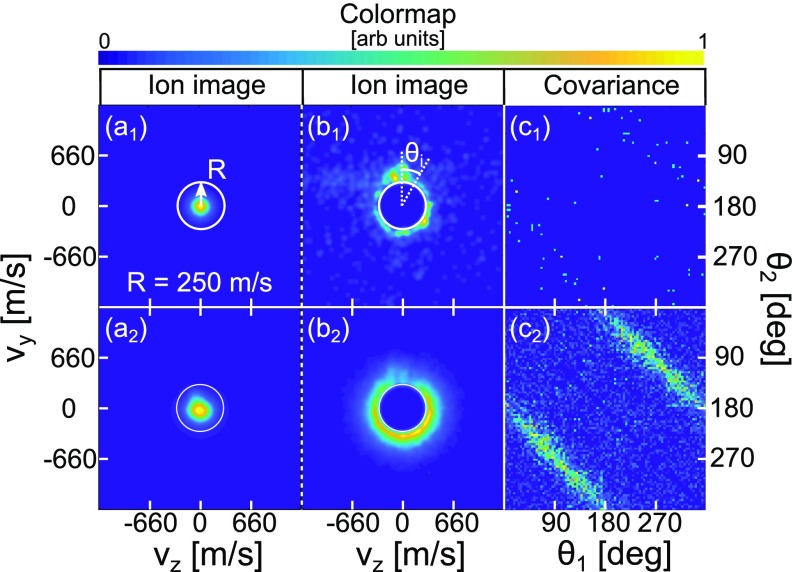
(a_1_) Tc^+^ ion image recorded for a low partial pressure of tetracene in the pickup cell (monomer doping condition); (a_2_) Tc^+^ ion image recorded for a higher partial pressure of tetracene in the pickup cell (dimer-doping condition); (b_1_)–(b_2_) The same image as (a_1_) and (a_1_) but with the center removed; (c_1_)–(c_2_) Corresponding angular covariance maps created from the ion count outside the white circles. The ion images are obtained without alignment, with the probe pulse at an intensity of 3 TW/cm^2^.

First, we show that it is possible to form and detect Tc dimers in the He droplets. The strategy is the same as that recently applied to identify CS_2_ and OCS dimers^25,26^ and relies on detecting kinetic Tc^+^ ions as a sign of dimers. In detail, if a droplet contains just one Tc molecule and this molecule is ionized by the probe pulse, the resulting Tc^+^ ion will have almost zero kinetic energy. In contrast, if a droplet contains a dimer and both of its monomers are singly ionized, the internal Coulomb repulsion of the Tc^+^ ions will cause them to gain kinetic energy. Figure 3(a_1_) shows a Tc^+^ ion image recorded with the probe pulse only. The ions are localized in the very center of the image with more than 98% of them having a velocity less than 250 m/s. These low-velocity ions are ascribed as originating from the ionization of singly-doped tetracene molecules. The ionization potential of tetracene is 6.97 eV, and the photon energy of the probe photons is 3.1 eV. Thus, we believe that ionization is the result of 3-photon absorption by the tetracene molecules.

Figure 3(a_2_) also shows a Tc^+^ ion image recorded with the probe pulse only but for a higher partial pressure of the tetracene gas in the pickup cell. The image is still dominated by an intense signal in the center, but now ions are also being detected at larger radii corresponding to higher velocities. This can be highlighted by cutting the center in the image. The images in Figs. 3(b_1_) and 3(b_2_) are the same as the images in Figs. 3(a_1_) and 3(a_2_), respectively, but with a central cut removing contributions of Tc^+^ ions with a velocity lower than 250 m/s. It is now clear that the image in Fig. 3(b_2_) contains a significant amount of ions away from the central part. We assign the high-velocity ions to ionization of both Tc molecules in droplets doped with a dimer.

To substantiate this assignment, we determined if there are correlations between the emission directions of Tc^+^ ions, implemented through covariance analysis. Let *X*^(*i*)^ be the discrete random variable that denotes the number of ions detected at an angle *θ_i_* with respect to the vertical centerline, see Fig. 3(b_1_). Experimentally, the detected ions are binned into *M* equal-size intervals over the 360° range, and thus, the angular distribution of the ions can be represented by the vector

$$X=\{X^{\left(1\right)},X^{\left(2\right)},\ldots X^{\left(M\right)}\}.$$ (1)

As mentioned in Sec. II the resulting ion images are averaged over a large number, *N*, of individual frames. In practice, the angular distribution is therefore given by the expectation value of **X**,

$$\langle X\rangle =\left\{\frac{1}{N}\sum_{n=1}^{N}x_{n}^{\left(1\right)},\frac{1}{N}\sum_{n=1}^{N}x_{n}^{\left(2\right)},\ldots ,\frac{1}{N}\sum_{n=1}^{N}x_{n}^{\left(M\right)}\right\},$$ (2)

where $x_{n}^{\left(i\right)}$ is the outcome (number of ions) of the random variable $X_{n}^{\left(i\right)}$ related to the angle *θ_i_* for the *n*th frame acquired. The covariance can now be calculated by the standard expression

Cov(X,X)=⟨XX⟩−⟨X⟩⟨X⟩, (3)

using the ions in the radial range outside of the annotated white circle. The result, displayed in Fig. 3(c_2_), is referred to as the angular covariance map. We used an angular bin size of 4° which gives *M *=* *90. The covariance map reveals two distinct diagonal lines centered at *θ*_2_ = *θ*_1_ ± 180°. These lines show that the emission direction of a Tc^+^ ion is correlated with the emission direction of another Tc^+^ ion departing in the opposite direction. This strongly indicates that the ions originate from ionization of both Tc molecules in dimer-containing droplets and subsequent fragmentation into a pair of Tc^+^ ions. Therefore, we interpret that the angular positions of the Tc^+^ ion hit outside the white circle as a measure of the (projected) emission directions of the Tc^+^ ions from dimers. Note that the angular covariance signal extends uniformly over 360°. This shows that the axis connecting the two Tc monomers is randomly oriented at least in the plane defined by the detector. This is to be expected in the absence of an alignment pulse. Also note that at the low partial pressure of tetracene, used for the image in Fig. 3(a_1_), the pronounced lines in the angular covariance map are no longer present, see Fig. 3(c_1_), indicating that there are essentially no dimers under these pickup conditions.

### B. Angular covariance maps for aligned tetracene dimers

Next, we carried out experiments aiming at determining the conformation of the Tc dimers. In the first set of measurements, described in this section, the dimers are aligned and then doubly ionized with the probe pulse, and the emission direction of the Tc^+^ ions is recorded. We then calculated their angular covariance maps, which were proven to provide useful information about the dimer conformation in the cases of CS_2_ and OCS dimers.^25,26^

Based on recent findings for similar-size molecular systems, we expect that the strongest degree of alignment occurs around the peak of the alignment pulse and that, upon truncation of the pulse, the degree of alignment lingers for 10–20 ps, thereby creating a time window where the alignment is sharp and the alignment pulse intensity reduced by several orders of magnitude.^48^ It is crucial to synchronize the probe pulse to this window because Tc^+^ ions are fragmented when created in the presence of the alignment pulse. The time-dependent measurements of the Tc^+^ yields, presented in Sec. IV D, show this effect explicitly. Consequently, the probe pulse is sent 10 ps after the peak of the alignment pulse as sketched in Fig. 1. In the previous experiments on CS_2_ and OCS dimers, the probe pulse was sent at the peak of the alignment pulse, and no truncation was needed, because both CS2^+^ and OCS^+^ ions can survive the alignment field.

**FIG. 4.**
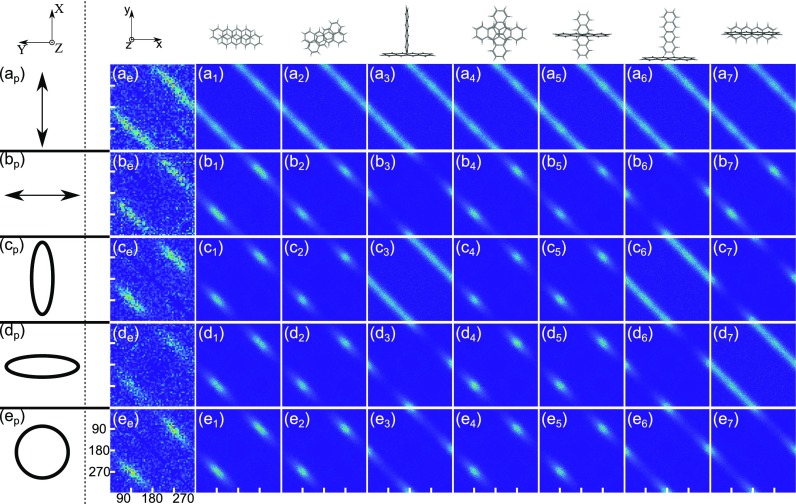
Covariance maps obtained for the Tc dimer. Indices a, b, c, d, and e refer to the alignment laser pulse polarization used as linear perpendicular, linear parallel, elliptical perpendicular, elliptical parallel, and circular, respectively, as shown in the first column with index p (the laboratory axes are illustrated at the top). Perpendicular and parallel refer to the angle between the main polarization axis and the plane of the detector. Index e (second column from the left) refers to the covariance maps obtained in the experiments, while indices 1–7 refer to different candidate conformations of the tetracene dimer depicted on top of the corresponding column. Each panel axis ranges linearly from 0 to 360^∘^.

The experimental results, recorded for different polarization states of the alignment pulse, are presented in the second column of Fig. 4. The partial pressure of tetracene vapor was set to the same value as that used for the data in row (2) in Fig. 3, i.e., under doping conditions where a significant number of the He droplets contain dimers. The Tc^+^ ion images are not shown, only the angular covariance maps created from the ions in the corresponding images originating from ionization of the dimers. In practice, these are the ions detected outside of a circle with the same diameter as the one shown in Fig. 3(a_2_). The intensity of the probe pulse was again 3 TW/cm^2^ for all the angular covariance maps shown in Fig. 4.

The different rows in Fig. 4 are the results of different polarization states of the alignment pulse and thus different spatial alignments of the dimers. In row (a), the alignment pulse was linearly polarized perpendicular to the detector plane, i.e., along the X axis—see Fig. 1. This induces 1D alignment with the most polarizable axis of the Tc dimer confined along the X axis. The covariance map [Fig. 4(a_e_)] contains two prominent stripes, centered at *θ*_2_ = *θ*_1_ ± 180°, very similar to those observed with the probe pulse only [Fig. 3(b_2_)]. This implies that the axis connecting the two Tc monomers is randomly oriented perpendicular to the X axis. Figure 4(b_e_) is also obtained for 1D aligned dimers but now the most polarizable axis, defined by the polarization direction of the alignment pulse, is confined along the Y axis, i.e., in the detector plane. It is seen that the covariance signal no longer extends over all angles but rather appears as two islands centered at (90°, 270°) and (270°, 90°), respectively. Panels (c_e_) and (d_e_) of Fig. 4 were recorded with an elliptically polarized alignment pulse with an intensity ratio of 3:1 in order to induce 3D alignment^49,50^ where the most polarizable axis of the dimer is confined along the major polarization axis [parallel to the X axis in panel (c) and to the Y axis in panel (d)] and the second most polarizable axis along the minor polarization axis [parallel to the Y axis in panel (c) and to the X axis in panel (d)]. The covariance maps also show islands localized around (90°, 270°) and (270°, 90°). Finally, the dimers were aligned with a circularly polarized alignment pulse, which confines the plane of the dimer to the polarization (X, Y) plane, but leaves it free to rotate within this plane. Again, the covariance signals are two islands localized around (90°, 270°) and (270°, 90°).

### C. Comparison of experimental covariance maps to simulated covariance maps

To identify possible conformations of the dimer that can produce the covariance maps observed, we simulated covariance maps for seven archetypal dimer conformations shown at the top of Fig. 4. The first two conformers are those predicted by our computational modeling to be stable in the gas phase, with the tetracene monomers parallel to each other and either parallel displaced (conformation 1) or slightly rotated (conformation 2). The other five conformations were chosen as representative examples of other possible geometries. Although the computational modeling does not predict them to be stable in the gas phase, they may get trapped in shallow local energy minima in the presence of the cold He environment as previously observed for, e.g., the HCN trimers and higher oligomers.^22,51^

The strategy we applied to simulate the covariance map for each of the dimer conformations is the following: (1) determine the alignment distribution, either 1D or 3D, of the dimer; (2) calculate the laboratory-frame emission angles of the Tc^+^ ions for each dimer conformation assuming Coulomb repulsion between two singly charged monomers using partial charges on each atomic center; (3) determine the angular distribution in the detector plane, **X**; and (4) calculate the covariance map, Cov(**X**, **X**) which can be compared to experimental findings. The details of each of the four steps of the strategy are outlined in the appendix.

Starting with the alignment pulse linearly polarized along the X axis [row (a)], all proposed conformers are found to produce stripes centered at *θ*_2_ = *θ*_1_ ± 180°. This is the same as in the experimental covariance map, making these covariance maps unable to distinguish between the proposed dimer structures. The second case is where the alignment pulse is linearly polarized along the Y axis [row (b)]. All conformations, except numbers 3 and 6, lead to covariance islands localized around (90°, 270°) and (270°, 90°) as in the experimental data. In contrast, conformations 3 and 6 lead to covariance islands localized around (0°, 180°) and (180°, 0°). Such covariance maps are inconsistent with the experimental observations and, therefore, conformations 3 and 6 can be discounted. To understand the covariance maps resulting from conformations 3 and 6, we note that their main polarizability components are *α_yy_* > *α_xx_* > *α_zz_* (see Table II). The linearly polarized alignment field will lead to alignment of the molecular y axis along the polarization axis (the Y axis). Upon Coulomb explosion, the two Tc^+^ ions will thus be ejected along the polarization axis of the alignment pulse, i.e., along 0° and 180°.

**TABLE II.** Polarizability tensors for each conformer presented in Fig. 4. The polarizability components are expressed in Å^3^ and are listed in the (x,y,z) order. The “Approximate” column comes from the summation of the polarizability tensor of each monomer. The “exact” column contains values resulting from DFT calculations. The results presented in Fig. 4 used the exact values.

Conformation	Polarizability tensors	
	Approximate	Exact
1	$\left(\begin{array}{ccc} 126.2 & 0 & 0\\ 0 & 47.9 & 0\\ 0 & 0 & 47.9 \end{array}\right)$	$\left(\begin{array}{ccc} 104.0 & -24.9 & 1.0\\ -24.9 & 64.5 & 1.5\\ 1.0 & 1.5 & 44.1 \end{array}\right)$
2	$\left(\begin{array}{ccc} 126.2 & 0 & 0\\ 0 & 47.9 & 0\\ 0 & 0 & 47.9 \end{array}\right)$	$\left(\begin{array}{ccc} 91.5 & -12.0 & 0.5\\ -12.0 & 78.6 & 0\\ 0.5 & 0 & 34.9 \end{array}\right)$
3	$\left(\begin{array}{ccc} 79.0 & 0 & 0\\ 0 & 79.0 & 0\\ 0 & 0 & 63.8 \end{array}\right)$	$\left(\begin{array}{ccc} 74.5 & 0 & 0\\ 0 & 97.3 & 0\\ 0 & 0 & 57.1 \end{array}\right)$
4	$\left(\begin{array}{ccc} 95.0 & 0 & 0\\ 0 & 95.0 & 0\\ 0 & 0 & 31.9 \end{array}\right)$	$\left(\begin{array}{ccc} 84.3 & 0 & 0\\ 0 & 84.3 & 0\\ 0 & 0 & 34.4 \end{array}\right)$
5	$\left(\begin{array}{ccc} 95.0 & 0 & 0\\ 0 & 79.0 & 0\\ 0 & 0 & 47.9 \end{array}\right)$	$\left(\begin{array}{ccc} 85.4 & 0 & 0\\ 0 & 74.0 & 0\\ 0 & 0 & 57.4 \end{array}\right)$
6	$\left(\begin{array}{ccc} 95.0 & 0 & 0\\ 0 & 79.0 & 0\\ 0 & 0 & 47.9 \end{array}\right)$	$\left(\begin{array}{ccc} 85.6 & 0 & 0\\ 0 & 94.1 & 0\\ 0 & 0 & 43.2 \end{array}\right)$
7	$\left(\begin{array}{ccc} 126.2 & 0 & 0\\ 0 & 47.9 & 0\\ 0 & 0 & 47.9 \end{array}\right)$	$\left(\begin{array}{ccc} 104.0 & 0 & 0\\ 0 & 41.6 & 0\\ 0 & 0 & 60.9 \end{array}\right)$

In row (c), the alignment pulse is elliptically polarized with the major (minor) polarization axis parallel to the X axis (Y axis). The covariance maps for conformations 1, 2, 4, and 5 show islands at (90°, 270°) and (270°, 90°) similar to the experimental data in Fig. 4(c_e_). In contrast, the covariance map for conformation 7 contains two islands centered at (0°, 180°) and (180°, 0°), and therefore, we discard this conformation among the candidates for the experimental observations. The polarizability components of conformation 7 are *α_xx_* > *α_zz_* > *α_yy_*. The elliptically polarized field will align the x axis along the X axis and the z axis along the Y axis. Following Coulomb explosion, the two Tc^+^ ions will thus be ejected along the minor polarization axis (the Y axis).

In row (d), the alignment pulse is elliptically polarized but now with the major (minor) polarization axis parallel to the Y axis (X axis). Again, the covariance maps for conformations 1, 2, 4, and 5 show islands at (90°, 270°) and (270°, 90°) similar to the experimental data in Fig. 4(d_e_). In contrast, the covariance islands for conformations 3, 6, and 7 differ from the experimental results. Since these three conformations have already been eliminated, the covariance maps in row (d) do not narrow further the possible candidates for the dimer conformation(s).

In row (e), the alignment pulse is circularly polarized. Once again, the covariance maps for conformations 1, 2, 4, and 5 show islands at (90°, 270°) and (270°, 90°) similar to the experimental data in Fig. 4(d_e_). Thus, the covariance maps resulting from molecules aligned with the circularly polarized pulse do not allow us to eliminate additional conformations of the tetracene dimer. At this point, we are left with conformations 1, 2, 4, and 5, which all present covariance maps consistent with the experimental data.

In the case of the OCS and CS_2_ dimers, an additional experimental observable, besides the parent ions, was available for further structure determination, namely, the S^+^ ion resulting from Coulomb fragmentation of the molecular monomers.^25,26^ Fragmentation of Tc will result in H^+^ or hydrocarbon fragments. Both H^+^ and hydrocarbon fragment ions can originate from different parts of the Tc molecule, and thus, their angular distributions may be less useful for extracting further structural information of the dimer than what was the case for the S^+^ ions in the previous studies of OCS and CS_2_. Instead, we performed an alternative type of measurements by recording the alignment-dependent ionization yield of the Tc dimer. As described in Sec. IV D, such measurements allow us to discount further conformations.

### D. Ionization anisotropy

Previous works, experimentally as well as theoretically, have shown that the rate of ionization of molecules induced by intense linearly polarized laser pulses depends strongly on the alignment of the molecules with respect to the polarization direction of the pulse.^52–55^ In this section, we use the alignment-dependent ionization rate of the tetracene dimers to infer further information about their possible conformation. The starting point is to characterize the alignment-dependent yield of Tc^+^ ions produced when the tetracene monomers are ionized by the probe pulse. In practice, this involves using the monomer doping condition, similar to that used for the data presented in Fig. 3, row (a), and, furthermore, analyzing only the low kinetic energy Tc^+^ ions stemming primarily from ionization of monomers. The measurements were performed with the alignment pulse linearly polarized either parallel or perpendicular to the probe pulse polarization and as a function of the delay between the two pulses.

**FIG. 5.**
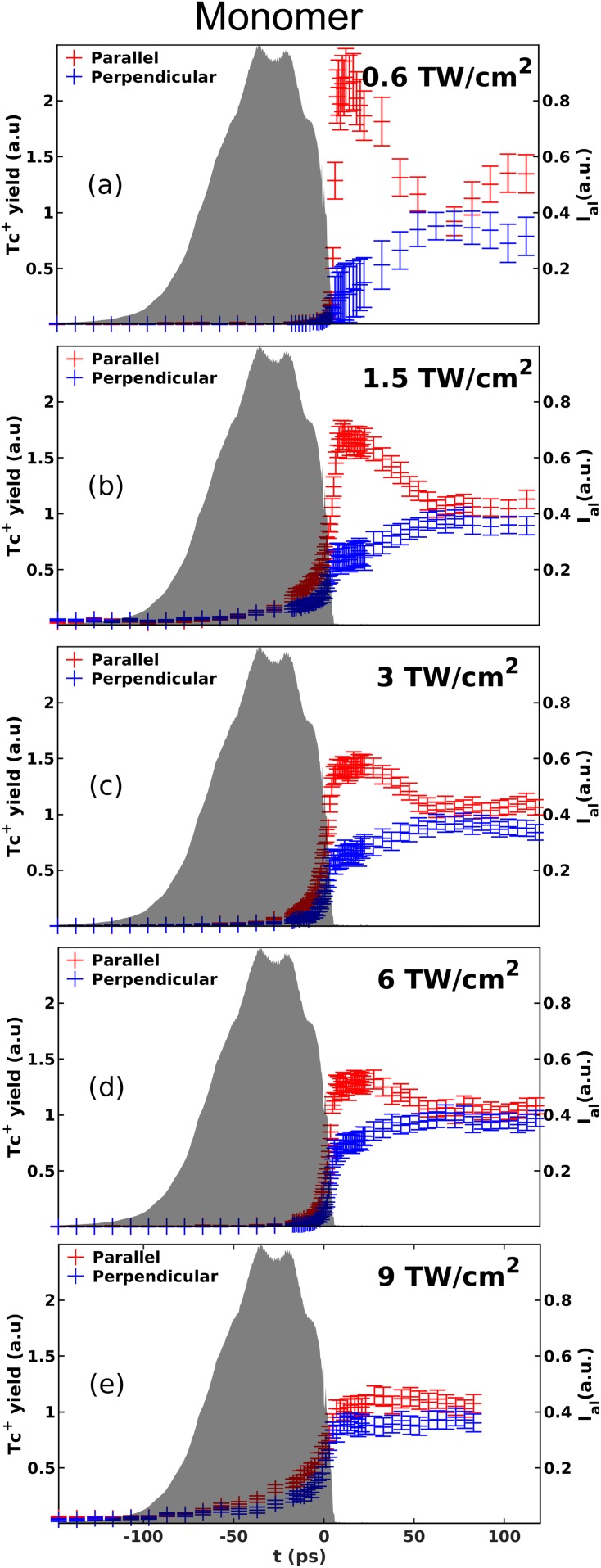
Time-dependent yield of Tc^+^ ions originating from ionization of 1D aligned tetracene molecules at five different probe laser pulse intensities written in bold inside each panel [(a)–(e)], with the linearly polarized probe pulse parallel (red) or perpendicular (blue) to the alignment pulse polarization. In each panel, the shaded area represents the intensity profile of the alignment pulse obtained by cross correlation with the probe laser pulse. In each panel, the Tc^+^ ion yield has been normalized to the mean of the yields obtained in the parallel and perpendicular geometries at times longer than 55 ps.

The results obtained for five different intensities of the probe pulse, I_probe_, are shown in Fig. 5. In all five panels, the Tc^+^ signal is very low, almost zero, when the ionization occurs while the alignment pulse is still on. The reason is that the Tc^+^ ions produced can resonantly absorb one or several photons from the alignment pulse, which will lead to fragmentation, i.e., destruction of intact tetracene parent ions. Previously, similar observations were reported for other molecules.^48,56^ To study the alignment-dependent ionization yield, using Tc^+^ ions as a meaningful observable, it is therefore necessary to conduct measurements after the alignment field is turned off. At *t *=* *10 ps, the intensity of the alignment pulse is reduced to 0.5% of its peak value. This is sufficiently weak to avoid the destruction of the Tc^+^ ions and, crucially, at this time, the degree of alignment is still expected to be almost as strong as at the peak of the alignment pulse.^48^ The red and blue data points show that for I_probe_ = 0.6 TW/cm^2^, the ionization yield is a factor of ∼7 times higher for the parallel compared to the perpendicular geometry. In other words, the cross section for ionization is higher when the probe pulse is polarized parallel rather than perpendicular to the long axis of the tetracene molecule. This observation is consistent with experiments and calculations on strong-field ionization of related asymmetric top molecules like naphthalene, benzonitrile, and anthracene.^54,57^

At longer times, the Tc^+^ signal for the parallel geometry decreases, reaches a local minimum around *t *=* *70 ps, and then increases slightly again, while the perpendicular geometry shows a mirrored behavior. This behavior is a consequence of the time-dependent degree of alignment induced when the alignment pulse is truncated.^48^ Finally, panels (b)–(e) of Fig. 5 show that the contrast between the Tc^+^ yield in the parallel and perpendicular geometries at *t *=* *10 ps decreases as I_probe_ is increased. We believe that this results from saturation of the ionization process.

**FIG. 6.**
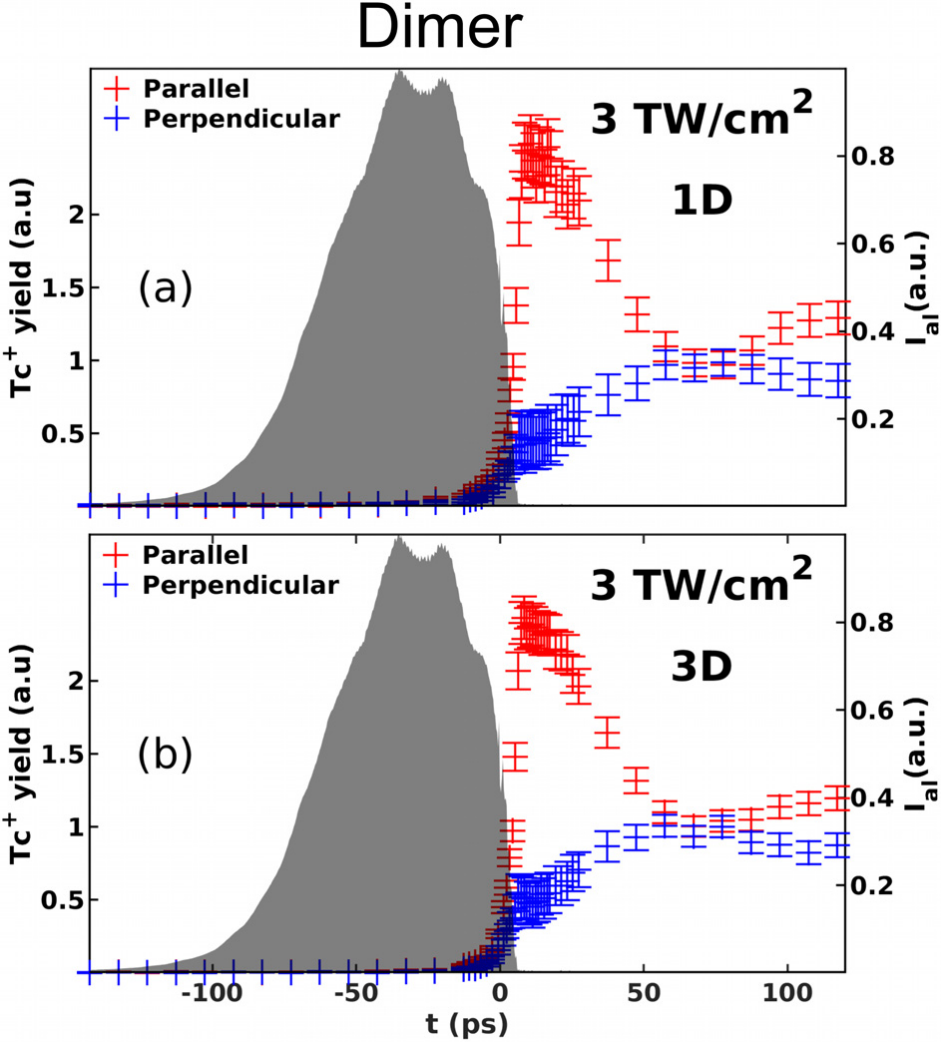
Time-dependent yield of Tc^+^ ions originating from ionization of (a) 1D aligned tetracene dimers; (b) 3D aligned tetracene dimers. The meaning of the perpendicular and parallel curves and the shaded areas is the same as in Fig. 5. In both panels, the Tc^+^ ion yield has been normalized to the mean of the yields obtained in the parallel and perpendicular geometries at times longer than 55 ps.

Next, similar measurements were conducted for the tetracene dimer by using the dimer doping condition, i.e., as for the data presented in Fig. 3 row (b), and analyzing only the high kinetic energy Tc^+^ ions stemming from ionization of dimers. The intensity of the probe pulse was set to 3 TW/cm^2^ rather than 0.6 TW/cm^2^, in order to obtain a sufficient probability for ionizing both Tc monomers in the dimers. Panel (a) shows the result for 1D aligned monomers. The time dependence of the Tc^+^ ion yield is very similar to that recorded for the Tc monomer at the same probe intensity, Fig. 5(c), for both the parallel and perpendicular polarization geometries. In fact, the ratio of the Tc^+^ ion yield in the parallel and perpendicular geometries at *t *=* *10 ps is ∼5.5, which is even larger than the ratio recorded for the monomer, ∼2.3. Such a significant difference in the ionization yield for the parallel and perpendicular geometries implies that the structure of the dimer must be anisotropic, as in the case of the monomer, i.e., possesses a “long” axis leading to the highest ionization rate when the probe pulse is polarized along it. Such an anisotropic structure is compatible with conformations 1 and 2 but not with conformations 4 and 5. The experiment was repeated for 3D aligned molecules. The results, displayed in Fig. 6(b), are almost identical to those obtained for the 1D aligned molecules. They corroborate the conclusion made for the 1D aligned case, but they do not allow us to distinguish between conformations 1 and 2.

### E. Discussion

The comparison of the experimental covariance maps to the calculated maps leaves us with conformations 1 and 2, the two lowest-energy structures predicted by our gas-phase calculations. We note that the rotated conformers obtained by DFT minimization may also possess an offset, where the two centers of charge do not lie on a common axis perpendicular to the molecular planes. Calculations yield offsets between the charge centers ranging between 0.9 and 1.3 Å depending on the method, conformers optimized with the quantum force field being almost symmetric with values below 0.01 Å. For comparison, the offset in the parallel displaced conformer is closer to 2 Å. The rotated conformers predicted here are thus also partly shifted. For the application of measuring singlet fission effects, this offset is essential, as perfect stacking of chromophores leads to a cancelation of the interactions singlet fission relies on.^4,34^ These results thus suggest that helium nanodroplets may be a fruitful route to exploring singlet fission processes.

The quantum chemistry calculations were carried out for isolated tetracene dimers. Although the interaction with helium was expected to be negligible in rationalizing the conformations of the Tc dimer, it may still influence the dynamical formation when the two tetracene molecules are picked up in the droplet. The helium solvent is known to be attracted more strongly to the hydrocarbon and somewhat freeze at its contact,^58^ possibly resulting in snowball precursors.^59^ Such effects may even be magnified in the presence of multiple molecules,^60^ and it is possible that commensurate conformers such as the parallel displaced structure mostly identified in our experiment may be kinetically favored once embedded in helium droplets.

As discussed in Sec. IV C, comparison of the experimental angular covariance maps for Tc^+^ to the calculated maps does not allow us to distinguish between the rotated-parallel and slipped-parallel conformations—and this would also be the case for the slipped-and-rotated-parallel conformation. However, if an atom, like F, was substituted for one of the hydrogens in each Tc molecule, then distinction between the conformations might become feasible by observing the relative angle between the emission of F^+^ ions from the two monomers. Previous experiments on halogen-atom substituted biphenyls in the gas phase have shown that angular covariance maps, generated from recoiling halogen ions following Coulomb explosion, are well suited for determination of bond angles and dihedral angles.^28,61^ We believe that transfer of this methodology to dimers of halogenated PAHs embedded in He droplets is feasible and promising for structure determination, including time-resolved measurements.

Distinction between the slipped-parallel and the parallel-slightly rotated structures could perhaps also be possible by a detailed measurement of the alignment-dependent ionization yield induced by a linearly polarized probe pulse. In practice, the dimers would be 3D aligned under field-free conditions, similar to the data presented in Fig. 6, and then the ionization yield recorded as a function of the angle between the probe pulse polarization and the major polarization axis of the elliptically polarized alignment pulse. For the slipped-parallel structure, the yield should peak rather sharply at 0°, whereas the maximum would be broader for the parallel-slightly rotated structure. It is even possible that such measurements could provide information about the angle between the two monomers in the parallel-slightly rotated structure.

## V. CONCLUSIONS

The purpose of this study was to obtain information about the conformation of tetracene dimers in a combined experimental and theoretical study. Experimentally, tetracene dimers were formed inside He nanodroplets. A strong femtosecond probe laser pulse was used to ionize both Tc molecules in the dimer, leading to a pair of recoiling Tc^+^ cations resulting from their internal Coulomb repulsion. These kinetic Tc^+^ ions provided an experimental observable uniquely sensitive to droplets doped with dimers. Next, a slow turn-on, fast turn-off, moderately intense laser was used to create a window of field-free alignment shortly after the pulse. Synchronizing the probe pulse to this window, the dimers, aligned either 1-dimensionally or 3-dimensionally, were Coulomb exploded and the covariance map of the emission directions of the Tc^+^ recoil ions determined. As a reference, angular covariance maps were calculated for seven different conformations including the two predicted to be the most stable by our quantum chemistry calculations and another five chosen as representative examples of other possible geometries. The experimental angular covariance maps were found to be consistent with four of the calculated maps. An additional dimer structure sensitive measurement was conducted, namely, how the yield of strong-field ionization depends on the polarization axis of the probe pulse with respect to the alignment of the dimer. It was found that the ionization yield is a factor of five times higher when the probe pulse polarization was parallel compared to perpendicular to the most polarizable axis of the dimer. This result is only consistent with the two tetracene molecules in the dimer being parallel to each other and either slightly displaced or slightly rotated. These are the two most stable gas-phase conformations of the dimer according to our quantum chemistry calculations.

## APPENDIX A: SIMULATION PROCEDURE

The identification of dimer conformations from the covariance maps relies on comparison with simulated maps predicted for different candidate structures. The individual tetracene monomers are expected to be rigid and the problem amounts to finding the relative orientations between the two molecules and the possible shift between their centers of mass.

For each conformation considered, covariance maps were generated by extracting the projection of the separation distance ($\left|{\vec{r}}_{\text{diff}}\right|$) between the center of mass of each tetracene molecule on the detector plane for different polarizations of the alignment laser pulse. The separation distance indicates the direction of recoil that the two Tc^+^ should follow by repelling each other. This section details the overall procedure.

## APPENDIX B: CONFORMATIONS

One important ingredient in explaining the observed covariance maps is the polarizability tensor of the tetracene dimer, which is responsible for its interaction with the laser pulses and is determined by its structure.

The polarizability of the tetracene monomer was calculated with a DFT method (wB97xD)^62^ using the diffuse basis set aug-pcseg-n,^38^ after a geometry optimization with a similar method but a more localized basis set pcseg-n.^63^ These calculations performed with the Gaussian09 software package^47^ yielded the polarizability components as *α_xx_* = 63.1 Å^3^, *α_yy_* = 31.9 Å^3^, and *α_zz_* = 15.9 Å^3^, where *x*, *y*, and *z* refer to the major, minor, and orthogonal axes of tetracene, respectively.

The polarizability tensor of the dimer is sometimes assumed to be the sum of the polarizability tensor of each molecule. However, this approximation usually underestimates the interaction polarizability along the axis connecting the molecules while overestimating it along the axes perpendicular to it.^64^ Simulations carried out for urea (CH_4_N_2_O) and fullerenes reported an increase in the polarizability component along the dimer axes by 9.9% and 17.8%, respectively, while the other components displayed a decrease by a few percents only.^64^ In our case, the anisotropy in the polarizability tensor of tetracene could be considered as sufficiently large that we can neglect these effects and assume the polarizability of the dimer to be a linear sum, although some caution should be taken with some of the conformations presented below. To ascertain this ambiguity, we calculated the exact polarizability for each conformation shown in Table II using a fixed geometry and a similar method and basis as the ones mentioned for the monomer. We see that using the exact polarizabilities breaks the symmetry that some of the dimers would possess if only the sum of their polarizabilities were considered. We thus only use the exact polarizabilities for the simulations presented above.

## APPENDIX C: LASER-INDUCED ALIGNMENT

After the diagonalization of the polarizability tensors obtained for each conformation, it is possible to calculate the potential energy surface when an electric field is applied. The potential can be evaluated using second-order perturbation theory^65^ and is defined as

$$V=-\frac{1}{2}{\vec{E}}^{T}\alpha_{\text{dimer}}\vec{E}=-\frac{1}{2}{\vec{\mu }}_{\mathrm{ind}}^{T}\cdot \vec{E},$$ (C1)

where the superscript T stands for transpose, $\alpha_{\text{dimer}}$ is the polarizability tensor of the conformer, ${\vec{\mu }}_{\mathrm{ind}}$ is the induced dipole moment, and $\vec{E}$ is the electric field applied on the system.

In our case, the electric field comes from a strong nonresonant laser pulse and can be expressed as

$${\vec{E}}_{\text{laser}}=\frac{E_{0}}{\sqrt{1+\epsilon^{2}}}\left(\begin{array}{c} \;\text{cos}\;\omega t\\ \pm \varepsilon \;\text{sin}\;\omega t\\ 0 \end{array}\right).$$ (C2)

In this equation, *E*_0_ is the peak amplitude of the electric field that we can extract from the experiment by measuring the intensity of the laser pulse, *ε* is the ellipticity parameter which ranges from 0 (linear polarization) to 1 (circular polarization), and *ω* is the laser frequency.

Usually, the Schrödinger equation is then solved and the distribution of rotational states that will be populated in the presence of the electric field is calculated to extract the overall angular distribution of the complex. However, in our approach, we drastically simplify the problem by making use of the adiabaticity of the alignment process, which will result in an angular confinement of the complex at the bottom of the potential well presented in Eq. (C1). The nonperfect alignment is accounted for by addition of a spread in the angular distribution.

To connect ${\vec{E}}_{\mathrm{laser}}$ in Eq. (C2) to the electric field $\vec{E}$ used in Eq. (C1), we need both ${\vec{E}}_{\mathrm{laser}}$ and $\alpha_{\text{dimer}}$ to be expressed in the same frame. In our case, we decided to make use of the diagonal form of the polarizability tensor in the molecular frame (MF) and to calculate the potential surface in this frame. For this purpose, we used a uniform distribution for all the possible directions of ${\vec{E}}_{\text{laser}}$ in the MF. A uniform sphere made of 10 000 direction vectors was created to represent the main axis of the polarization ellipse. This would be sufficient for a linearly polarized pulse but not for an elliptically polarized pulse, where the minor axis has to be included. In this case, for each major axis orientation, 1000 angular steps of the minor axis were used.

To make the procedure explicit and to give a more intuitive form for the interaction potential, we develop the expression using a general form for ${\vec{E}}_{\text{laser}}$ in the molecular frame,

$${\vec{E}}_{\text{laser}}=\frac{E_{0}}{\sqrt{1+\varepsilon^{2}}}\left(\begin{array}{c} a_{x}\;\text{cos}\;\omega t+b_{x}\varepsilon \;\text{sin}\;\omega t\\ a_{y}\;\text{cos}\;\omega t+b_{y}\varepsilon \;\text{sin}\;\omega t\\ a_{z}\;\text{cos}\;\omega t+b_{z}\varepsilon \;\text{sin}\;\omega t \end{array}\right),$$ (C3)

where *a_i_* and *b_i_* are coefficients fulfilling the conditions

$$\sum a_{i}^{2}=\sum b_{i}^{2}=1,$$ (C4)

$$\sum a_{i}b_{i}=0.$$ (C5)

These coefficients will depend on the unitary transformations that have been applied to ${\vec{E}}_{\text{laser}}$ and will be the sum of trigonometric functions with the angles related to the applied rotations. Implementing this expression in Eq. (C1) and developing it using the long duration of the pulse compared to the optical frequency, we obtain

$${\left\langle V\right\rangle }_{T}=-\frac{E_{0}^{2}}{4\left(1+\varepsilon^{2}\right)}\sum_{i=x,y,z}\left(a_{i}^{2}+b_{i}^{2}\varepsilon^{2}\right)\alpha_{\mathrm{ii}}.$$ (C6)

This shape is interesting since only square quantities appear in the summation. To develop the expression a bit further, some assumptions are needed about the shape of the polarizability tensor. Assuming that two of its components are larger than the last one (*α_xx_*, *α_yy_* ≫ *α_zz_*), the minimum of the potential will be found if one tries to maximize the coefficients *a_x_*_,__*y*_ and *b_x_*_,__*y*_, which will naturally lead to put *a_z_* = *b_z_* = 0 owing to the condition of Eq. (C4). A form consistent with the previous requirements would then be

$$\left(\begin{array}{c} a_{x}\\ a_{y}\\ a_{z} \end{array}\right)=\left(\begin{array}{c} \;\text{cos}\;\phi \\ -\text{sin}\;\phi \\ 0 \end{array}\right),\;\left(\begin{array}{c} b_{x}\\ b_{y}\\ b_{z} \end{array}\right)=\left(\begin{array}{c} \;\text{sin}\;\phi \\ \;\text{cos}\;\phi \\ 0 \end{array}\right),$$ (C.7)

thus giving

$${\left\langle V\right\rangle }_{T}=-\frac{E_{0}^{2}}{8}\left[\left(\alpha_{\mathrm{xx}}+\alpha_{\mathrm{yy}}\right)+\left(\alpha_{\mathrm{xx}}-\alpha_{\mathrm{yy}}\right)\frac{1-\varepsilon^{2}}{1+\varepsilon^{2}}\text{cos}\;2\phi \right].$$

In the extreme case of *ε* = 0 (linear pulse), the minimum of *V* is achieved when *ϕ* = 0[*π*] if *α_xx_* > *α_yy_* or *ϕ* = *π*/2[*π*] if *α_yy_* > *α_xx_*. With *ε* = 1 (circular pulse) or if *α_xx_* = *α_yy_*, the dependence on *ϕ* disappears, leaving an isotropic distribution in the plane (all *ϕ* are allowed).

## APPENDIX D: FRAME CONNECTION

Picking the orientation of the electric field that gives the lowest potential energy, the minima are chosen using a spread (Δ*E*_lim_) based on the temperature of the dimers inside the helium droplets (0.37 K). The energy spread Δ*E*_lim_ is chosen to include 99% of the Boltzmann populations, which gives Δ*E*_lim_ = 0.15 meV. Only energies fulfilling *E* < *E*_min_ + Δ*E*_lim_ are selected, with *E*_min_ being the lowest possible value in the potential energy surface.

For each orientation found above, a rotation matrix connecting the initial frame (IF) to the laboratory frame (LF) can be defined, labeled $R_{\text{IFtoLF}}$. This matrix is generated by combining two rotation matrices: the first one connects the IF where the dimer has been described to the MF where its polarizability tensor is diagonal and the second one is generated by finding the rotation needed to project the orientation of the electric field (both major and minor axes) in the LF where it should be fixed as in the experiment. These two rotations applied on the IF allow us to express the interdimer distance in the LF.

## APPENDIX E: PROJECTION

The direction of the repulsion vector is calculated from the resulting Coulombic force,

$${\vec{F}}_{C}^{\text{IF}}=\sum_{i}\sum_{j}q_{i}q_{j}\frac{{\vec{r}}_{i}-{\vec{r}}_{j}}{|{\vec{r}}_{i}-{\vec{r}}_{j}{|}^{3}},$$ (E.1)

where *q_i_* and *q_j_* are the partial charges on each atom (shown in Fig. 2), ${\vec{r}}_{i}$ and ${\vec{r}}_{j}$ are their respective positions, and the summation is carried out to consider the combination of all pairs between the two molecules. Its direction in the laboratory frame is given from the vector ${\vec{F}}_{C}^{\text{LF}}=R_{\text{IFtoLF}}{\vec{F}}_{\text{C}}^{\text{IF}}$. Since multiple solutions are possible, a random selection over 100 000 molecules, using a Boltzmann weight, was used to choose which $R_{\text{IFtoLF}}$ to apply to ${\vec{F}}_{C}^{\text{IF}}$. To take into account imperfect alignment, the direction vector of ${\vec{F}}_{C}^{\text{LF}}$ was then rotated using a conical distribution described by a polar angle *ψ* picked from a Gaussian distribution with standard deviation *σ_ψ_* = *π*/6. This corresponds to an alignment distribution of ${\vec{F}}_{C}^{\text{IF}}$ characterized by $\langle \;{\text{cos}}^{2}\psi \rangle =0.8$.

The vector ${\vec{F}}_{C}$ is then projected onto the detector plane leading to two solutions, referring to the detector plane being parallel to the main axis of the polarization or perpendicular to it. This is represented in Fig. 1 with the main polarization axis of the alignment pulse being similar to the Y axis and the minor axis to the X axis or the main axis to the X axis and the minor to the Y axis, respectively. For each solution, an angle *θ*_2D_ is extracted by projecting it onto the polarization axis parallel to the detector plane. A maximal velocity can be estimated from the magnitude of the separation vector ($\left|{\vec{F}}_{C}\right|$) based on the resulting Coulomb repulsion between the partial charges,

$$V_{\text{Coulomb}}=\sum_{i}\sum_{j>i}\frac{q_{i}q_{j}}{2|{\vec{r}}_{i}-{\vec{r}}_{j}|}=K=\frac{mv_{\text{max}}^{2}}{2},$$

hence,

$$\;|{\vec{v}}_{\text{max}}|=\sqrt{\frac{2}{m}V_{\text{Coulomb}}}$$ (E.2)

with *K* being the kinetic energy, *m* the mass of the molecule, and *v*_max_ the maximum velocity that the system can acquire upon Coulomb repulsion. The Coulomb potential has been divided by two to take into account the symmetric behavior of the two molecules during Coulomb explosion. The initial distance between the molecules will depend on the conformations considered. A minimal distance of 3.5 Å was assumed with the centers of mass based on the molecular modeling in Sec. III except for geometries 3 and 6 where a larger separation had to be added (∼6 Å) to avoid unphysical overlap. Owing to the screening of low kinetic energy ions during the experiment (black circle area in Fig. 3), a filter can be applied on the velocity *v*, and values below *v*_lim_ are excluded. In our case, *v*_lim_ = 0.25 mm/*μ*s as estimated from simulations with SIMION^66^ giving the expected velocities as a function of the spectrometer radius.

## APPENDIX F: COVARIANCE

For a given dimer conformation, the covariance map is eventually generated using the angle *θ*_2D_ coming from the projection. This angle is selected with a uniform distribution weighted by its number of occurrences, as explained in Appendix E. For each selected angle $\theta_{2D}^{i}$, a mirror angle $\theta_{2D}^{j}=\theta_{2D}^{i}+180^{{}^{\circ}}$ is generated as well to account for the recording of two molecules emitted upon Coulomb dissociation of a given dimer and producing two counts. A Gaussian noise with standard deviation $\sigma_{\theta_{2D}}$ was also applied on each angle to take into account the nonaxial recoil.^67^ Here, $\sigma_{\theta_{2D}}$ was extracted from experimental data by measuring the width in the covariance stripes originating from unaligned dimers.^68^ Using this procedure, we found $\sigma_{\theta_{2D}}\approx 15{}^{\circ}$.
